# Effect of 1 Year of Qigong Exercise on Cognitive Function Among Older Chinese Adults at Risk of Cognitive Decline: A Cluster Randomized Controlled Trial

**DOI:** 10.3389/fpsyg.2020.546834

**Published:** 2020-10-30

**Authors:** Jing Jin, Yin Wu, Shaohua Li, Suhui Jin, Lin Wang, Jian Zhang, Chenglin Zhou, Yong Gao, Zhen Wang

**Affiliations:** ^1^ School of Psychology, Shanghai University of Sport, Shanghai, China; ^2^ Department of Kinesiology, Boise State University, Boise, ID, United States; ^3^ School of Martial Arts, Shanghai University of Sport, Shanghai, China; ^4^ Shanghai Caoyang Middle School, Shanghai, China; ^5^ School of Kinesiology, Shanghai University of Sport, Shanghai, China

**Keywords:** qigong, exercise, cognitive function, cognitive decline, aging, elderly

## Abstract

**Background:** The rapidly aging Chinese population is showing an increase in age-related illnesses, including mild cognitive impairment and Alzheimer disease. The best types of physical activity for the improvement of cognition remain unknown. This study aimed to compare the effectiveness of a tailored qigong exercise with that of stretching exercise in the maintenance of cognitive abilities in Chinese elders at risk of cognitive decline.

**Methods:** Seventy-four community-dwelling adults aged ≥60 years were screened for eligibility. Using a randomized control group design, participants with scores ≥19 on the Chinese version of the Montreal Cognitive Assessment-Basic (MoCA) were allocated to a 1-year qigong intervention (*n* = 33) and a stretching control exercise group (*n* = 33). The primary outcome was the MoCA score, as a measure of global cognitive function, and secondary outcomes were globe cognition and five domain scores on the Chinese version of the Repeatable Battery for the Assessment of Neuropsychological Status (RBANS). The MoCA and RBANS were administered at baseline and 1 year after intervention to assess the effect of the exercises on cognitive decline.

**Results:** Twenty-five of 33 (75.8%) participants in the qigong group and 26 of 33 (78.8%) participants in the control group completed the 1-year exercise programs. A bivariate test revealed strong correlation between MoCA and RBANS total scores after the intervention (*r* = 0.517, *p* < 0.01). Generalized estimating equations revealed a lower risk of progression of cognitive decline at 1 year in the qigong group than in the control group (odds ratio, 0.314; 95% confidence interval, 0.103–0.961; *p* = 0.04). Two-way repeated-measures ANOVA followed by *post hoc t* tests with Bonferroni corrections indicated that MoCA and RBANS scores were significantly higher in the qigong group than in the control group (MoCA and RBANS global cognition, memory, visuospatial/constructional ability, and language, all *p* < 0.01), with the exception of RBANS attention score (*p* > 0.05).

**Conclusions:** One year of qigong practice was significantly superior to stretching exercise not only for the prevention of cognitive decline progression, but also for the improvement of several cognitive functions, among older Chinese adults at risk of cognitive decline.

## Introduction

The Chinese population is aging rapidly, bringing an inevitable increase in illnesses related to aging, including cognitive decline and impairment (World Health Organization, 2015), and more specifically mild cognitive impairment (MCI) (Langa and Levine, 2014) and Alzheimer disease (Gao et al., 2019). This shift is of great public-health and economic concern worldwide (Wimo et al., 2017; Gao et al., 2019). No specific pharmacologic therapy for these conditions can yet be recommended, as the efficacy of such treatments has not been demonstrated adequately (Massoud et al., 2007; Arvanitakis et al., 2019). Nonpharmacological interventions, including cognitive training, physical exercise, and non-invasive brain stimulation, are promising approaches to improve cognition during healthy and pathological aging (Cespon et al., 2018). Physical activity, a promising behavioral strategy, can improve cognitive function (Angevaren et al., 2008; Stillman and Erickson, 2018) and protect against cognitive decline (Sofi et al., 2011), especially MCI (Petersen et al., 2018). The most useful type of activity, and whether everyone benefits similarly from the same type of intervention, however, remains unknown (Miller et al., 2012).

Mind-body exercises, a slow, low-intensity type of physical activity, is particularly suitable for older adults (Jahnke et al., 2010; Guo et al., 2016). Qigong is a prime example of such exercise and can improve the cognitive performance of older adults with and without cognitive impairment (Zhang et al., 2018; Klein et al., 2019; Xia et al., 2019; Zheng et al., 2020). Growing evidence indicates that they reduce the levels of inflammatory markers, such as C-reactive protein and interleukin-6, with a larger pooled effect size than that for meditation (Morgan et al., 2014). Qigong involves the performance of a static (stationary) or dynamic set of meditative exercises with the intention of coordinating one’s mental energy, breathing, and physical movement (Guo et al., 2016). It requires less physical and cognitive effort than more complex practices and is a broad area of practice; qigong intervention protocols can be varied widely to benefit different aspects of cognitive performance (Guo et al., 2018; Chan et al., 2019). Qigong interventions have been shown to benefit mental health, with improvements in global cognition, thinking operations, brain processing speed, memory function, and attention (Tsang et al., 2013; Ladawan et al., 2017; Tao et al., 2017a,b; Myers et al., 2019; Xia et al., 2019). Although these results are promising, the potential benefit of qigong in cognitive function domains such as language, attention, and immediate/delayed memory have not been explored thoroughly. In addition, no high-quality randomized controlled trial (RCT) has examined the effects of qigong practice among older adults with and without cognitive impairment.

Age-related cognitive decline is defined as the development of typical performance decrements in various domains, including memory, processing speed, and attention (Salthouse, 1996; Glisky, 2007). Although this decline is normal, it has implications for problem solving, accuracy, and the speed of execution of everyday cognitive activities (Borella et al., 2017). In addition, the rate of decline is predictive of the development of clinical cognitive impairment (Plassman et al., 2010; Petersen, 2011). Neuropsychological tests have been developed to assess overall cognitive function and performance in specific domains (Smith et al., 2010). The Montreal Cognitive Assessment-Basic (MoCA) test is used to assess global cognitive function (Bernstein et al., 2011), and the Repeatable Battery for the Assessment of Neuropsychological Status (RBANS) is used to assess globe cognition and performance in five cognitive domains (Randolph et al., 1998).

In the present study, we aimed to determine the effect of tailored qigong exercise relative to that of stretching exercise on the cognitive functions, assessed using the MoCA test and RBANS, among older Chinese adults at risk of cognitive decline. Our primary hypothesis for this cluster RCT was that qigong would be more effective than stretching exercise in terms of the delay of age-related cognitive decline and improvement of several domains of cognitive function in our elderly population.

## Materials and Methods

### Participants

In March–April 2018, community-dwelling individuals were voluntarily recruited from a community center in the Wujiaochang area of Shanghai, China. All participants had good physical and functional abilities and no evidence of substantial cognitive impairment and were living independently at the time of enrollment. Study eligibility criteria were: (1) Chinese ethnicity, (2) age 60–80 years, (3) Around >1 year formal education, and (4) willingness to be randomly allocated to an intervention or control condition and to complete the 12-month intervention or control exercise. Exclusion criteria were: (1) participation in daily and/or structured qigong or other mind-body exercise for ≥15 min or muscle-strengthening activities (e.g., weight lifting) on 2 or more days a week in the previous 6 months; (2) severe cognitive impairment (MoCA score < 19; Chen et al., 2016); and (3) major medical or physical condition recorded on a case report form or reported by a healthcare provider that preclude exercise (e.g., high blood pressure, diabetes, obesity, cancer, brain tumor, stroke, malnutrition, major depressive disorder, schizophrenia, current chemotherapy/radiotherapy, disability or difficulty in hearing, vision, or communication).

Individuals who responded to the study recruitment materials were invited to present at a research facility for a detailed, face-to-face intake process, including consent form signing and completion of the MoCA and other baseline measures. Prior to providing written informed consent, participants were given sufficient time in a private room to ask questions about the study protocol and qigong exercise. Research assistants who were trained and monitored by the first author performed the screening and outcome assessments. The study was approved by the Ethics Advisory Committee at Shanghai University of Sport, China. Written informed consent was obtained from all participants before their inclusion in the study.

### Study Design

The observation period for this single-blind cluster RCT was 1 year. The eligible participants were randomly assigned to intervention group and control group by a computer-generated randomization. The group randomization was separated into opaque envelopes. This procedure was conducted by an independent researcher. Another researcher opened the envelope and proceeded with allocation. Treatment allocation was revealed to the participants after collection of baseline outcomes. The intervention group received qigong training, while the control group performed stretching exercise. Comprehensive cognitive function assessments were conducted at baseline and 1 year after intervention.

### Intervention and Control Exercises

The 12-month intervention was conducted in two phases. The induction phase comprised 2–3 weeks of exercise instruction, with regular weekly sessions held at the training centers until the participants were familiar with the logistics of exercises. During this period, participants had access to the centers for practice. In the maintenance phase, the participants were given videos of the programs to which they had been allocated on CD. The participating training centers provided facilities and arranged practice sessions for participants with the videos. The qigong masters provided monthly refresher lessons to both study groups, to boost adherence and ensure correct performance.

**Figure 1:**
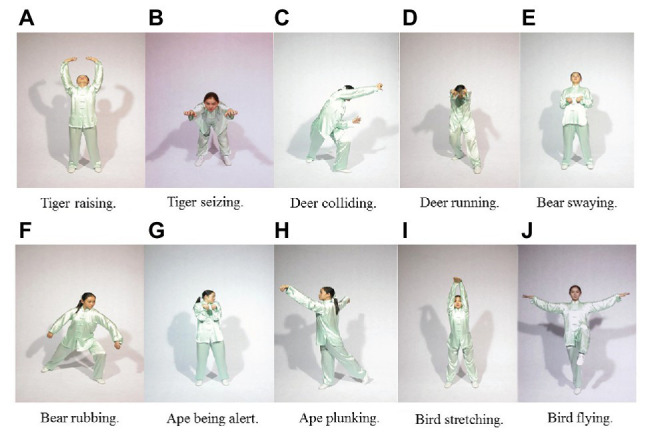
Ten movements of the qigong exercise (selected illustrations). **(A)** Tiger raising; **(B)** Tiger seizing; **(C)** Deer colliding; **(D)** Deer running; **(E)** Bear swaying; **(F)** Bear rubbing; **(G)** Ape being alert; **(H)** Ape plunking; **(I)** Bird stretching; **(J)** Bird flying.

The qigong intervention involved the practice of 10 core exercises (two movements imitating each of five animals; Guo et al., 2018): (1) tiger raising and seizing, (2) deer colliding and running, (3) bear swaying and rubbing, (4) ape being alert and plunking, and (5) bird stretching and flying (Figure 1). Four sets (~11 min/set) were performed in every exercise time, with each set includes two repetitions of each of the 10 exercise. Each practice set included musical accompaniment with the videos. The intervention also involved a static (stationary) or dynamic set of meditative exercises that is particularly suitable for and appealing to older adults (Guo et al., 2016, 2018). The qigong practice emphasized synchronized breathing, controlled weight shifting, mediation, and coordinated eye-head-hand movements with self-initiated (proactive) and self-induced (reactive) movement perturbations. The stretching exercise, serving as an exercise control group, consisted primarily of breathing, stretching, and relaxation activities, with most performed in a seated position.

The intervention and control exercises were performed on ≥2 days a week in ≥60-min sessions (10–15 min warm-up, 40–45 min core exercises, 5 min cool-down). During the core exercises, participants wore heart rate telemeters (RS800XSD; POLAR, Finland) and exercise intensity was controlled so that their heart rates remained in the range of (220 – age) × (60–80%). Most participants in the control group practiced in groups at the training centers; only two practiced alone being supervised remotely and kept exercise logs. In addition, all participants were asked to keep their original physical activity habits.

We defined serious adverse events (SAE) as death or the development of medical conditions requiring ≥1 day of hospitalization. All SAEs occurring during the 12-month study period were reported to the Ethics Advisory Committee at Shanghai University of Sport. No SAE or other serious event related to the intervention occurred during the study period.

### Measures

#### MoCA Test

The original English MoCA test (Bernstein et al., 2011; available at www.mocatest.org for clinical use) was translated to Chinese, with subtle linguistic and cultural modifications. The MoCA test is administered over about 10 min and has 30 items covering nine cognitive domains (executive function, language, orientation, calculation, conceptual thinking, memory, visuospatial perception, attention, and concentration). Total scores range from 0 to 30, with higher scores indicating better global cognitive function. The MoCA test is most suitable for the quantification of cognitive impairment (Bernstein et al., 2011), and has been shown to be reliable for MCI screening among Chinese elderly adults of all educational levels, with a high degree of acceptance and good reliability (Chen et al., 2016).

#### Repeatable Battery for the Assessment of Neuropsychological Status

The RBANS (Randolph et al., 1998) consists of 12 subtests administered over 30–40 min that yield five age-adjusted index scores (immediate memory, visuospatial/constructional, language, attention, and delayed memory), as well as a total cognitive function score. The RBANS is designed to evaluate disease progression and intervention efficacy, and has been shown to be sensitive for the detection and characterization of abnormal cognitive decline in older adults (Randolph et al., 1998). The original RBANS has shown high degrees of reliability and validity in elderly community populations (Duff et al., 2005), and the Chinese version has shown good reliability and validity in a community-dwelling older adult population (Cheng et al., 2011).

### Statistical Analysis

Baseline characteristics (e.g., age, sex, educational level) and cognitive ability (MoCA and RBANS scores) were compared between groups using the independent-sample *t* tests to identify differences for matching at baseline. The independent-sample *t* test was also used to detect difference in MoCA scores between males and females in each group at baseline and 1 year after intervention. Bivariate correlation of MoCA scores and RBANS total scores was examined. Using a cutoff MoCA score of 26 (Nasreddine et al., 2005), the number of participants with no cognitive impairment and MCI at baseline and 1 year after intervention was determined. Then, a generalized estimating equation was used to account for the effect of the intervention relative to the control on cognitive decline. The effects of the exercises on cognitive function were examined using two-way repeated-measures ANOVA, with the group (qigong, stretching) serving as the between-subject factor and time (baseline, 1 year) serving as the within-subject factor. *Post hoc* independent and paired *t* tests, respectively, were conducted with Bonferroni correction to examine differences between groups and time points. The SPSS software (version PASW Statistics 18, SPSS Inc.) was used for all statistical analyses, with the significance threshold set at *p* < 0.05.

## Results

### Participants and Baseline Characteristics

**Figure 2:**
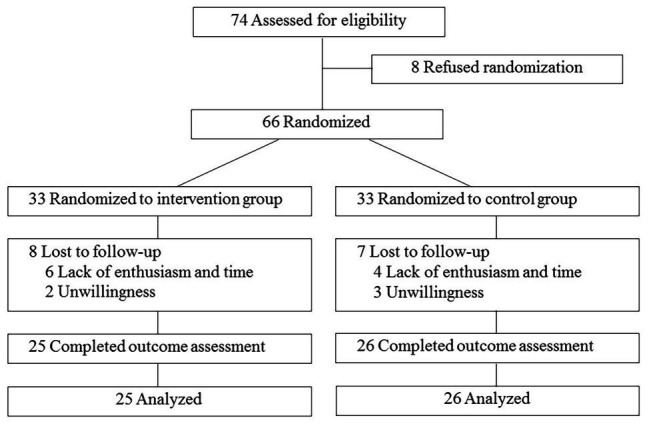
Flowchart showing participant recruitment and follow-up status.

Of 74 individuals screened, 66 were enrolled and randomized, while eight subjects refused to participate prior to randomization. Thirty-three individuals were randomly assigned to each group. The reasons given for refusal in the study included lack of enthusiasm and time and unwillingness to attend the training sessions. Fifty-one participants (25 in the intervention group, 26 in the control group) completed the exercise programs. The flow of study participants is illustrated in Figure 2.

Baseline characteristics and cognitive profiles were no significant differences in age, sex, educational level, MoCA score, and RBANS score between two groups at baseline (Table 1). The mean (SD) age of the participants was 66.1 (4.4) years; participants had a mean of 11.0 (2.2) years of education, and 43 (83.3%) were female.

**Table 1:** Participant characteristics and cognitive profiles at baseline.

Characteristic	All	Qigong group	Control group	*p* value
Number	51	25	26	ND
Sex, No. (%)				0.14
Male	8 (15.7)	2 (8.0)	6 (23.1)	
Female	43 (83.3)	23 (92.0)	20 (76.9)	
Age, mean (SD), y	66.1 (4.4)	66.7 (4.5)	66.4 (4.3)	0.57
Education level, mean (SD), y	11.0 (2.2)	11.1 (2.3)	10.9 (2.2)	0.67
Cognitive profile, mean (SD), score				
MoCA	23.8 (2.4)	23.7 (2.8)	23.8 (2.0)	0.85
RBANS total	177.9 (13.6)	175.1 (13.9)	180.7 (13.0)	0.14
Immediate memory	33.2 (5.9)	32.6 (6.7)	33.8 (4.8)	0.47
Visuospatial/Constructional	30.7 (4.2)	30.3 (3.5)	31.2 (4.8)	0.46
Language	27.0 (3.4)	26.8 (3.9)	27.2 (2.9)	0.71
Attention	46.6 (8.9)	45.2 (10.3)	48.0 (7.3)	0.25
Delayed memory	40.4 (4.8)	40.3 (4.8)	40.4 (4.8)	0.83

MoCA, Montreal Cognitive Assessment-Basic, Chinese version; ND, not determined; RBANS, Repeatable Battery for the Assessment of Neuropsychological Status.

### MoCA Score

**Figure 3:**
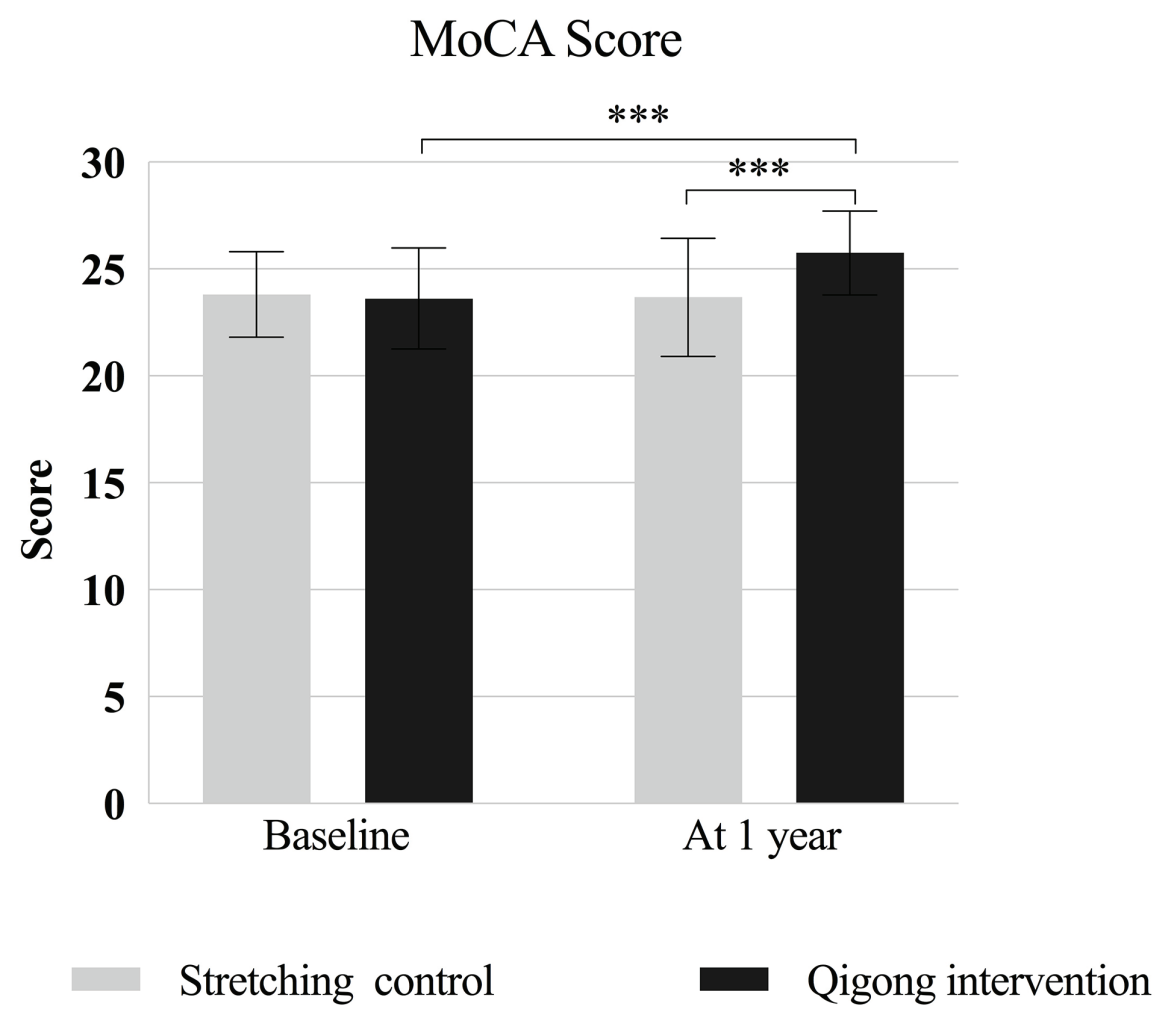
MoCA scores at baseline and at 1-year follow-up for the qigong intervention and stretching control group. MoCA, Montreal Cognitive Assessment-Basic, Chinese version. ^***^ *p* < 0.001 for the indicated comparisons.

For the MoCA score, two-way repeated-measures ANOVA revealed a significant main effect of time [*F*_(1,49)_ = 13.256, *p* = 0.001] and a significant interaction between time and group [*F*_(1,49)_ = 19.208, *p* < 0.001], but no significant main effect of group [*F*_(1,49)_ = 2.939, *p* = 0.09]. *Post hoc* analyses showed that the MoCA score at 1 year was significantly higher in the qigong group than in the control group (*p* = 0.001). The MoCA score increased significantly between baseline and 1 year in the qigong group (*p* < 0.001), but not in the control group (Figure 3). MoCA scores did not differ significantly between males and females in each group at baseline and 1 year.

### Rate of Cognitive Decline

The intervention group contained six (24.0%) individuals with no cognitive impairment at baseline and 14 (56.0%) individuals with no such impairment at 1 year; these numbers were 6 (23.1%) and 3 (11.5%), respectively, in the control group (Table 2). The numbers of participants with MCI at baseline and 1 year were 19 (76.0%; 95% CI, 0.549–0.906) and 11 (44.0%; 95% CI, 0.244–0.651), respectively, in the intervention group and 20 (76.9%; 95% CI, 0.564–0.910) and 23 (88.5%; 95% CI, 0.698–0.976), respectively, in the control group. Generalized estimating equations controlled for baseline differences revealed a lower risk of MCI development at 1 year in the intervention group than in the control group (intention-to-treat analyses: odds ratio, 0.314; 95% CI, 0.103–0.961, *p* = 0.04).

**Table 2:** Rate of progression to mild cognitive impairment among participants from baseline to the 1-year follow-up.

Cognitive status	Participants, No, (%)			
	Qigong group (*n* = 25 completers)		Control group (*n* = 26 completers)	
	Baseline	At 1 year	Baseline	At 1 year
No impairment	6 (24.0)	14 (56.0)	6 (23.1)	3 (11.5)
MCI (MoCA score < 26)	19 (76.0)	11 (44.0)	20 (76.9)	23 (88.5)

MCI, mild cognitive impairment; MoCA, Montreal Cognitive Assessment-Basic, Chinese version.

### RBANS Scores

#### Global Cognitive Ability

**Figure 4:**
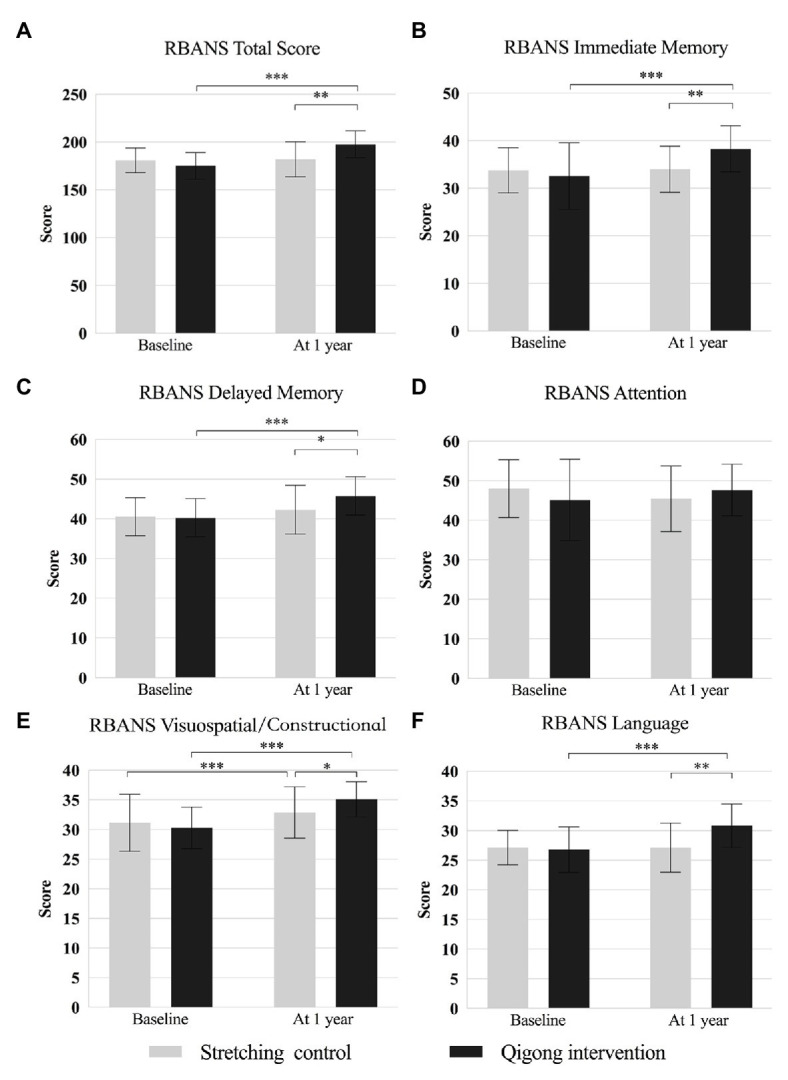
Comparison of the RBANS total and index scores between baseline and 1-year follow-up in the qigong intervention and stretching control group. **(A)** RBANS Total Score; **(B)** RBANS Immediate Memory; **(C)** RBANS Delayed Memory; **(D)** RBANS Attention; **(E)** RBANS Visuospatial/Constructional; **(F)** RBANS Language. RBANS indicates Repeatable Battery for the Assessment of Neuropsychological Status. ^*^ *p* < 0.05, ^**^ *p* < 0.01, and ^***^ *p* < 0.001 for the indicated comparisons.

For the RBANS total score, two-way repeated-measures ANOVA revealed a significant main effect of time [*F*_(1,49)_ = 39.100, *p* < 0.001] and significant interaction between time and group [*F*_(1,49)_ = 32.310, *p* < 0.001], but no significant main effect of group [*F*_(1,49)_ = 1.892, *p* = 0.18]. *Post hoc* analyses showed that this score was significantly higher in the intervention group than in the control group (*p* = 0.001) at 1 year. The RBANS total score was significantly higher at 1 year than at baseline in the intervention group (*p* < 0.001), but not in the control group (*p* = 0.69; Figure 4A). The MoCA and RBANS total scores correlated significantly at baseline (*r* = 0.500, *p* < 0.001) and 1 year (*r* = 0.517, *p* < 0.001).

#### Memory

For the RBANS immediate and delayed memory indices, two-way repeated-measures ANOVA revealed significant main effects of time [*F*_(1,49)_ = 15.983 and 23.296, respectively; both *p* < 0.001] and significant interactions between time and group [*F*_(1,49)_ = 13.600 and 6.362, *p* = 0.001 and *p* = 0.02, respectively], but no significant main effect of group [*F*_(1,49)_ = 1.335 and 1.648, *p* = 0.25 and *p* = 0.20, respectively]. *Post hoc* analyses showed that means of these scores were significantly higher in the intervention group than in the control group at 1 year (*p* = 0.003 and *p* = 0.03, respectively). The scores were significantly at 1 year than at baseline in the intervention group (both *p* < 0.001), but not in the control group (both *p* = 0.83; Figures 4B, C).

#### Attention

For the RBANS attention index, two-way repeated-measures ANOVA revealed significant interaction between time and group [*F*_(1,49)_ = 4.434, *p* = 0.04], but no significant main effect of time [*F*_(1,49)_ = 0.001, *p* = 0.98] or group [*F*_(1,49)_ = 0.028, *p* = 0.87]. *Post hoc* analyses showed no significant group difference (Figure 4D).

#### Visuospatial/Constructional Abilities

For the RBANS visuospatial/constructional index, two-way repeated-measures ANOVA revealed a significant main effect of time [*F*_(1,49)_ = 32.920, *p* < 0.001] and significant interaction between time and group [*F*_(1,49)_ = 7.271, *p* = 0.01], but no significant main effect of group [*F*_(1,49)_ = 0.475, *p* = 0.49]. *Post hoc* analyses showed that the mean of this score was significantly higher in the intervention group than in the control group at 1 year (*p* = 0.04). It was significantly higher at 1 year than at baseline in the intervention group (*p* < 0.001), but not in the control group (*p* = 0.69; Figure 4E).

#### Language Ability

For the RBANS language index, two-way repeated-measures ANOVA revealed a significant main effect of time [*F*_(1,49)_ = 11.849, *p* = 0.001] and significant interaction between time and group [*F*_(1,49)_ = 12.309, *p* = 0.001], but no significant main effect of group [*F*_(1,49)_ = 3.971, *p* = 0.05]. *Post hoc* analyses showed that mean of this score was significantly higher in the intervention group than in the control group at 1 year (*p* = 0.001). It was significantly higher at 1 year than at baseline in the intervention group (*p* < 0.001), but not in the control group (*p* = 0.96; Figure 4F).

## Discussion

This study showed that 1 year of qigong exercise delayed cognitive decline and promoted cognitive functions more effectively than did 1 year of stretching exercise among community-dwelling older Chinese adults at risk of cognitive decline.

Our MoCA score findings are in line with those of systematic reviews and meta-analyses, which have shown decreased age-related cognitive decline in older adults practicing qigong than in those participating in other interventions (Guo et al., 2016, 2018; Cheng, 2018; Zou et al., 2018; Chan et al., 2019). We found that the RBANS total score correlated strongly with the MoCA score, meaning that the RBANS is effective and valid for the assessment of cognitive function. Our findings for the RBANS domain scores extend the current body of knowledge based on RBANS indices, providing new evidence for the effectiveness of qigong interventions in the improvement of cognitive functions among older adults.

The precise mechanism(s) underlying the improvement in cognitive functions measure in this study is unclear. The breathing exercise combined with mental concentration and relaxation that is performed during qigong practice may help to improve cardiorespiratory and autonomic nervous system functions, which might in turn contribute to improvements in cognitive performance (Ladawan et al., 2017). Given the relationship between physical and mental health, general improvements in physical health or reductions of chronic disease symptoms may help to improve mental health (Abbott and Lavretsky, 2013). The combined physical and mental challenges tax physiological and neurophysiological processes, driving positive adaptations in the brain. Qigong has been shown to benefit specific aspects of cognitive functioning in frail elderly adults (Tsang et al., 2013). Previous studies showed the effects of qigong practice among older adults have focused on global cognitive function (Tsang et al., 2013; Ladawan et al., 2017; Tao et al., 2017a), memory function (Tao et al., 2017a,b) or attention (Xia et al., 2019). In this study, the tailored qigong intervention had stronger effects not only on global cognition, but also immediate/delayed memory, visuospatial/constructional ability, and language (but not attention) than did the control exercise.

Attention, which is highly sensitive to age-related decline (Braver and Barch, 2002), is a cognitive domain unrelated to memory that is impaired in the early stages of Alzheimer disease (Posner and Petersen, 1990) and deteriorates before other functions in patients with MCI who display significant declines in reaction time (Saunders and Summers, 2011). Attention can be enhanced by various methods, including the awareness of breathing and the body parts that is commonly involved in meditation and mindfulness training (Tang et al., 2007; Kozasa et al., 2012). Eight weeks of qigong training effectively improved attention in middle-aged participants, but these improvements disappeared 12 weeks after qigong practice cessation (Ladawan et al., 2017). Twenty-four weeks of mind-body exercise (60 min three times per week) was shown to improve attention in elderly adults with MCI (Xia et al., 2019). In our study, the RBANS attention index did not differ significantly between groups at baseline or 1 year but tended to be higher after 1 year of qigong practice and lower after 1 year of the control exercise. With very early age-related cognitive deficits, attention decline may be very serious; thus, significant improvement of this cognitive aspect may require lengthier qigong practice, although the intensity of qigong may not sufficient to significantly improve attention. Future studies could examine whether more rapid qigong movements (i.e., decreased reaction time) improve the attention aspect of cognitive function.

To our knowledge, this RCT is the first to evaluate the effectiveness of 1 year of qigong exercise on the cognitive function of older Chinese adults at risk of cognitive decline using the RBANS. Although substantial communication gaps exist between clinicians and community service providers, we have provided evidence that qigong, a mind-body exercise, is accessible and effectively delays some aspects of cognitive dysfunction among community-dwelling older adults.

This study has several limitations. First, the sample was small which could have reduced the accuracy of the results (Wittes, 2002). The participants in this study were screened by eligibility and exclusion criteria, and it is difficult to let the participants maintain 1-year qigong intervention. Second, the sample was predominantly female. We detected no sex-based difference in MoCA score between two groups, but biological sex is important variable when identifying optimal exercise prescriptions for the maintenance of cognitive health (Barha et al., 2020). Thus, future studies with a larger, sex-balanced samples are needed to further confirm the findings of our study. Third, we used stretching exercise as the control. Additional studies in which the control conditions are more comparable to the intervention, such as those consisting of another type of traditional Chinese exercise (e.g., tai chi, baduanjin), aerobic exercise (e.g., walking) or resistance exercise, may be needed to validate our findings.

## Conclusion

Among community-dwelling older Chinese adults at risk of cognitive decline, 1 year of qigong exercise was associated with decreased progression of cognitive decline and improved cognitive function relative to 1 year of stretching exercise.

## Data Availability Statement

The datasets generated for this study are available on request to the corresponding authors.

## Ethics Statement

The studies involving human participants were reviewed and approved by The Ethical Committee of Shanghai University of Sport (No. 2016013). The patients/participants provided their written informed consent to participate in this study. Written informed consent was obtained from the individual for the publication of any potentially identifiable images or data included in this article.

## Author Contributions

JJ conceived and designed the experiments, performed the experiments, analyzed the data, contributed reagents/materials/analysis tools, wrote the paper, prepared figures and/or tables, and reviewed drafts of the paper. YW analyzed the data, prepared figures and/or tables, and reviewed drafts of the paper. SJ and SL performed the experiments and contributed reagents/materials/analysis tools. LW and JZ reviewed drafts of the paper. YG analyzed the data, prepared figures and/or tables, and reviewed drafts of the paper. CZ and ZW conceived and designed the experiments and reviewed drafts of the paper. All authors contributed to the article and approved the submitted version.

### Conflict of Interest

The authors declare that the research was conducted in the absence of any commercial or financial relationships that could be construed as a potential conflict of interest. Written informed consent was obtained from the Yuanyuan Lv (she was an undergraduate in school of martial arts, Shanghai University of Sport) for the publication of any potentially identifiable images or data included in this article.
